# Association between Apolipoprotein E Gene Polymorphism and the Risk of Coronary Artery Disease in Chinese Population: Evidence from a Meta-Analysis of 40 Studies

**DOI:** 10.1371/journal.pone.0066924

**Published:** 2013-06-24

**Authors:** Yan-Wei Yin, Qian-Qian Sun, Bei-Bei Zhang, Ai-Min Hu, Hong-Li Liu, Qi Wang, Zhi-Zhen Hou

**Affiliations:** 1 Department of Emergency, Chinese PLA Air Force General Hospital, Beijing, P.R. China; 2 Jinsong Sanatorium of Beijing Air Force, Beijing, P.R. China; 3 Department of Medical Affairs, General Hospital of PLA Chengdu Military Area Command, Chengdu, P.R. China; “Mario Negri” Institute for Pharmacological Research, Italy

## Abstract

**Background:**

Epidemiological studies have evaluated the association between apolipoprotein E (ApoE) gene polymorphism and coronary artery disease (CAD) risk which developed inconsistent conclusions. To derive a more precise estimation of the relationship in Chinese population, we performed this meta-analysis.

**Methods:**

Databases, including PubMed, EMbase, Web of Science, CBMdisc and CNKI, were searched to get the genetic association studies. Additionally, hand searching of the references of identified articles were performed. All the statistical tests were performed using Review Manager 5.1.2 and Stata 11.0.

**Results:**

We identified a total of 40 studies, including 4,564 CAD cases and 3,985 controls. The results showed evidence for significant association between ApoE ε4 allele and CAD risk (for ε2/ε4 vs. ε3/ε3: OR = 1.86, 95% CI = 1.42–2.43, p<0.00001; for ε3/ε4 vs. ε3/ε3: OR = 2.34, 95% CI = 2.07–2.65, p<0.00001; for ε4/ε4 vs. ε3/ε3: OR = 2.89, 95% CI = 1.87–4.47, p<0.00001; for ε4 allele vs. ε3 allele: OR = 2.11, 95% CI = 1.91–2.35, p<0.00001).

**Conclusions:**

The present meta-analysis suggests an association between ApoE ε4 allele and increased risk of CAD in Chinese population. However, due to the small sample size in most of the included studies and the selection bias existed in some studies, the results should be interpreted with caution.

## Introduction

Coronary artery disease (CAD) is the most common cardiovascular disease, and its incidence is increasing in the developing countries. Despite it is well established that a poor diet, advanced age, smoking, hypertension, diabetes, and dyslipidemia are associated with increased risk of CAD, a detailed etiology underlying CAD is still obscure. Recently, numerous molecular epidemiological studies have focused on the association between apolipoprotein E (ApoE) gene polymorphism and CAD risk, and indicated that ApoE ε4 allele exerts an important role in the development of CAD.

ApoE is a plasma protein involved in the lipid metabolism and participates in the transports of cholesterol and triglyceride [1]–[3]. The human ApoE gene is on chromosome 19q13.2, with three common variant alleles: ε2, ε3 and ε4. Every individual inherits one allele of ApoE from each of their parents, which yields six possible genotypes: ε2/ε2, ε2/ε3, ε2/ε4, ε3/ε3, ε3/ε4, and ε4/ε4 [4]. ApoE gene polymorphism have been found to affect ApoE gene transcription and the serum levels of cholesterol and triglyceride [5], [6], thus changing the progression of atherosclerosis, which is the main underlying pathology of CAD. Recently, a variety of molecular epidemiological studies have focused on the relationship between ApoE gene polymorphism and CAD risk. However, results in different studies have been inconsistent. A meta-analysis of worldwide studies including 48 studies has provided evidence that the ε4 allele of ApoE was a risk factor for the development of CAD [7]. Considering that potential ethnic difference might be associated with the distribution of genotypes, we conducted a meta-analysis by collecting and sorting the previously published studies in Chinese population.

## Materials and Methods

### Literature Search

This meta-analysis followed the Preferred Reporting Items for Systematic Reviews and Meta-analyses (PRISMA) criteria [8]. We searched all published studies (up to November 28, 2012) in databases of PubMed, Embase, Web of Science, Cochrane Library, Chinese Biomedical Literature analysis and retrieval system for compact disc (CBMdisc) and China National Knowledge Infrastructure (CNKI). These computer searches were limited to English and Chinese language articles, but did not include reviews and editorials. The following keywords were used for searching: “apolipoprotein E” OR “ApoE” AND “polymorphism” OR “mutation” OR “variant” OR “variation” OR “genotype” AND “coronary artery disease” OR “CAD” OR “coronary heart disease” OR “CHD” OR “ischemic cardiovascular disease” OR “atherosclerosis”. The equivalent Chinese terms were used in the Chinese databases. Additionally, hand searches for related articles were also performed.

### Inclusion Criteria

The inclusion criteria for identified articles were as follows: (1) studies on the relationship between ApoE gene polymorphism and CAD; (2) case-control studies using either a hospital-based or a population-based design; (3) studies with full text articles; (4) sufficient data for estimating an odds ratio (OR) with 95% confidence interval (CI); (5) not republished data.

### Data Extraction

Information was carefully extracted from all eligible publications independently by two authors(Yin YW and Hu AM) of this article. Disagreement was resolved by consensus. If these two authors could not reach a consensus, the result was reviewed by a third author (Sun QQ). For each study, information was extracted including first author, year of publication, area of study population, source of controls, total numbers of cases and controls, genotyping methods, and distribution of genotypes and alleles in cases and controls, respectively. In addition, evidence of Hardy-Weinberg equilibrium was also collected (HWE, p<0.05 of HWE was considered significant).

### Quality Score Assessment

To determine the methodological quality of each study, we used the Newcastle-Ottawa scale, which uses a “star” rating system to judge the quality of observational studies [9]. The NOS ranges between zero (worst) up to nine stars (best). Studies with a score equal to or higher than seven were considered to be of high quality. Two authors (Liu HL and Wang Q) independently assessed the quality of included studies, and the result was reviewed by a third author (Hou ZZ). Disagreement was resolved by discussion.

### Statistical Analysis

The strength of association between ApoE gene polymorphism and CAD risk was measured by ORs with 95% CIs. The combined ORs were respectively calculated for seven genetic models (ε2/ε2 vs. ε3/ε3, ε2/ε3 vs. ε3/ε3, ε2/ε4 vs. ε3/ε3, ε3/ε4 vs. ε3/ε3, ε4/ε4 vs. ε3/ε3, ε2 allele vs. ε3 allele and ε4 allele vs. ε3 allele). The fixed-effects model was used in the absence of between-study heterogeneity, Otherwise, a random effects model was adopted [10]. Heterogeneity was assessed by the Q-test and I^2^ statistic, p<0.10 and I2>50% indicated evidence of heterogeneity [11], [12]. Galbraith plot was used to detect the potential sources of heterogeneity. Sensitivity analysis was conducted by limiting the meta-analysis to studies conforming to HWE. Publication bias was analyzed by Begg’s funnel plot and Egger’s regression test (p<0.05 was considered representative of statistically significant publication bias) [13]. All above statistical analyses were performed using Review Manager 5.1.2 (Cochrane Collaboration, The Nordic Cochrane Centre, Copenhagen) and Stata 11.0 (StataCorp LP, College Station, TX).

## Results

### Study Characteristics

**Figure 1:**
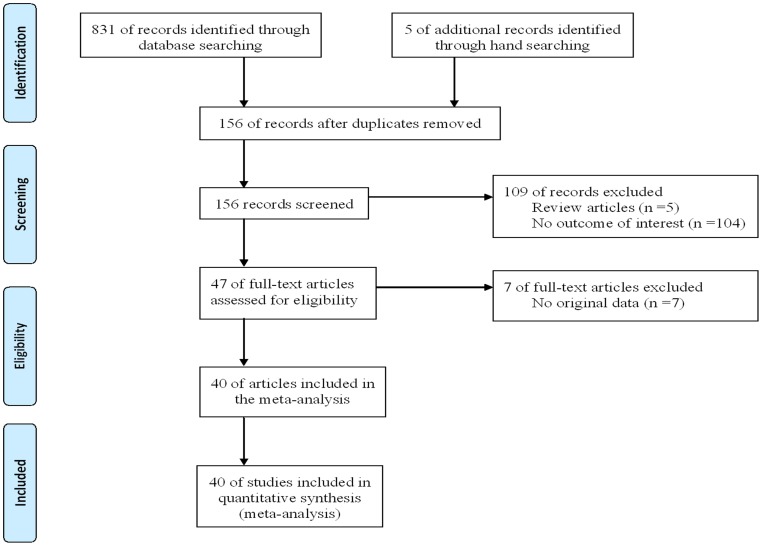
Flow diagram of the study selection process.

The present study met the PRISMA statement requirements (Table S1 and Fig. 1). A total of 40 studies were included in the final meta-analysis according to the inclusion criteria [14]–[53], containing 4,564 CAD cases and 3,985 controls. Table 1 and Table 2 show the studies identified and their main characteristics. These eligible studies were from 17 provinces of China including Anhui, Beijing, Fujian, Gansu, Guangdong, Heilongjiang, Hubei, Hunan, Jiangsu, Jilin, Shandong, Shanghai, Sichuan, Tianjin, Xinjiang, Yunnan and Zhejiang. Controls were population-based in 34 studies [14]–[30], [32]–[39], [42]–[46], [48]–[50], [52], hospital-based in six studies [31], [40], [41], [47], [51], [53]. There were 10 studies did not follow the HWE [17], [26], [29], [31], [37], [39], [46], [47], [49], [51]. The NOS results showed that the average score was 7.85, which indicated that the methodological quality was generally good.

**Table 1:** Characteristics of studies included in this meta-analysis.

						Genotypes distribution (case/control)										
First author	Year	GL	GM	SOC	SS(case/control)	ε2/ε2	ε2/ε3	ε3/ε3	ε2/ε4	ε3/ε4	ε4/ε4	ε2	ε3	ε4	HWE Y/N(P)	Score
Zhu [14]	1997	Beijing	PCR- RFLP	PB	100/43	0/0	9/5	80/33	0/0	11/5	0/0	9/5	180/76	11/5	Y(0.863)	8
Zhang [15]	1998	Beijing	PCR-RFLP	PB	96/130	0/2	11/16	67/91	3/0	14/21	1/1	14/20	159/219	19/23	Y(0.266)	8
Cao [16]	1999	Heilongjiang	PCR-RFLP	PB	78/85	0/0	7/11	61/68	1/0	9/6	0/0	8/11	138/153	10/6	Y(0.789)	8
Zhang [17]	2000	Zhejiang	PCR-RFLP	PB	61/71	1/0	2/5	46/56	1/3	10/6	1/1	5/8	104/123	13/11	N(0.007)	7
Wu [18]	2000	Shanghai	PCR-RFLP	PB	114/135	0/0	15/18	72/101	6/4	20/12	1/0	21/22	179/232	28/16	Y(0.065)	8
Li [19]	2000	Gansu	PCR-RFLP	PB	95/46	1/1	6/4	61/34	3/1	21/6	3/0	11/7	149/78	30/7	Y(0.340)	9
Zhang [20]	2001	Guangdong	PCR- RFLP	PB	71/69	0/0	4/8	46/53	5/1	16/7	0/0	9/9	112/121	21/8	Y(0.810)	8
Bai [21]	2001	Guangdong	PCR-RFLP	PB	50/47	0/0	4/5	40/39	0/0	6/3	0/0	4/5	90/86	6/3	Y(0.939)	9
Zhang [22]	2001	Shanghai	PCR-RFLP	PB	46/25	0/0	7/1	28/20	1/1	9/3	1/0	8/2	72/44	12/4	Y(0.169)	8
Peng [23]	2001	Guangdong	PCR-RFLP	PB	213/180	0/0	29/27	123/126	5/3	53/24	3/0	34/30	328/303	64/27	Y(0.436)	8
Pan [24]	2001	Shanghai	PCR-RFLP	PB	100/50	2/1	7/6	69/38	1/0	21/5	0/0	12/8	166/87	22/5	Y(0.532)	8
Zhu [25]	2002	Shandong	PCR-RFLP	PB	60/30	0/0	7/4	44/15	0/1	8/9	1/1	7/5	103/43	10/12	Y(0.956)	8
Yang [26]	2003	Xinjiang	PCR- RFLP	PB	124/70	3/3	6/13	71/35	3/8	40/11	1/0	15/27	188/94	45/19	N(0.036)	7
Li [27]	2003	Jiangsu	PCR-RFLP	PB	129/90	0/0	20/12	73/68	5/2	29/8	2/0	25/14	195/156	38/10	Y(0.424)	8
Peng [28]	2003	Hunan	PCR-RFLP	PB	150/157	1/1	21/13	93/122	1/1	30/18	4/2	24/16	237/275	39/23	Y(0.424)	9
Liu [29]	2003	Guangxi	PCR-RFLP	PB	120/121	1/0	18/21	75/88	5/2	16/8	5/2	25/23	184/205	31/14	N(0.028)	8
Li [30]	2003	Hubei	PCR-RFLP	PB	125/116	1/0	17/20	64/81	4/1	38/13	1/1	23/21	183/195	44/16	Y(0.616)	7
Cao [31]	2003	Jilin	PCR-RFLP	HB	37/72	0/0	5/9	28/56	2/4	2/3	0/0	7/13	63/124	4/7	N(0.000)	7
Zhu [32]	2003	Jiangsu	PCR-RFLP	PB	204/136	1/1	18/12	153/106	0/1	24/15	8/1	20/15	348/239	40/18	Y(0.738)	8
Sun [33]	2004	Jilin	PCR-RFLP	PB	96/113	0/0	13/19	51/78	1/2	30/13	1/1	14/21	145/188	33/17	Y(0.678)	9
Liao [34]	2004	Shandong	PCR-RFLP	PB	90/90	0/0	5/10	58/71	6/1	19/8	2/0	11/11	140/160	29/9	Y(0.817)	8
Zhang [35]	2004	Shandong	PCR-RFLP	PB	120/121	1/0	15/20	63/83	3/2	37/14	1/2	20/22	178/200	42/20	Y(0.354)	8
Pan [36]	2005	Yunnan	PCR-RFLP	PB	98/87	1/0	14/14	51/60	1/1	31/11	0/1	17/15	147/145	32/14	Y(0.743)	8
Wu [37]	2005	Fujian	PCR-RFLP	PB	341/86	22/0	60/12	83/56	64/5	102/13	10/0	168/17	328/137	186/18	N(0.047)	7
Ou [38]	2005	Beijing	PCR-RFLP	PB	200/100	1/0	28/17	98/66	1/1	67/14	5/2	31/18	291/163	78/19	Y(0.399)	7
Xiang [39]	2005	Hubei	PCR-RFLP	PB	77/63	1/1	6/4	42/48	0/1	24/7	4/2	8/7	114/107	32/12	N(0.031)	8
Ji [40]	2005	Sichuan	PCR-RFLP	HB	56/30	0/0	5/4	38/23	2/1	11/2	0/0	7/5	92/52	13/3	Y(0.439)	8
Feng [41]	2005	Tianjin	PCR- RFLP	HB	68/70	1/0	8/10	43/52	3/1	13/7	0/0	13/11	107/121	16/8	Y(0.823)	9
Wang [42]	2006	Hubei	PCR-RFLP	PB	201/360	0/3	28/46	118/263	2/1	52/45	1/2	30/53	316/617	56/50	Y(0.444)	7
Ma [43]	2006	Shandong	PCR-RFLP	PB	88/75	0/0	18/9	47/55	11/0	8/11	4/0	29/9	120/130	27/11	Y(0.620)	8
Wang [44]	2007	Hunan	PCR-RFLP	PB	30/30	0/1	3/3	18/20	2/2	7/4	0/0	5/7	46/47	9/6	Y(0.176)	7
Chu [45]	2007	Heilongjiang	PCR-RFLP	PB	328/220	7/0	58/41	162/140	9/5	92/34	0/0	81/46	474/355	101/39	Y(0.170)	8
Wang [46]	2008	Jilin	PCR- RFLP	PB	50/113	0/0	5/14	37/88	1/4	7/7	0/0	6/18	86/197	8/11	N(0.005)	8
Zhang [47]	2008	Tianjin	PCR- RFLP	HB	100/100	2/4	12/15	54/67	0/0	30/13	2/1	16/23	150/162	34/15	N(0.041)	7
Sun [48]	2008	Liaoning	PCR-RFLP	PB	50/156	0/0	7/21	31/118	2/3	9/14	1/0	9/24	78/271	13/17	Y(0.306)	7
Huang [49]	2009	Guangxi	PCR- RFLP	PB	93/100	5/12	3/19	46/49	6/9	27/11	6/0	19/52	122/128	45/20	N(0.001)	8
Hong [50]	2009	Guangdong	PCR-RFLP	PB	97/35	0/0	14/6	51/24	1/1	30/4	1/0	15/7	146/58	33/5	Y(0.791)	7
Shi [51]	2009	Anhui	PCR- RFLP	HB	98/110	0/0	4/3	44/71	12/9	36/27	2/0	16/12	128/172	52/36	N(0.000)	7
Hu [52]	2009	Hubei	PCR-RFLP	PB	251/200	0/0	30/28	148/138	3/5	67/29	3/0	33/33	393/333	76/34	Y(0.208)	8
Qi [53]	2010	Shandong	PCR- RFLP	HB	59/53	3/1	8/8	32/34	1/1	15/8	0/1	15/11	87/84	16/11	Y(0.850)	9

GL: geographical location; GM: genotyping methods; SS: sample size; HWE: Hardy-Weinberg equilibrium, Y: yes, N: no.

PCR-RFLP: polymerase chain reaction restriction fragment length polymorphism.

SOC: source of controls; PB: population-based; HB: hospital-based.

**Table 2:** Results of meta-analysis for ApoE gene polymorphism and risk of CAD.

		ε2/ε2 vs ε3/ε3		ε2/ε3 vs ε3/ε3		ε2/ε4 vs ε3/ε3		ε3/ε4 vs ε3/ε3		ε4/ε4 vs ε3/ε3		ε2 allele vs ε3 allele		ε4 allele vs ε3 allele	
Category	SS(case/control)	OR(95% CI)	P_Q_	OR(95% CI)	P_Q_	OR(95% CI)	P_Q_	OR(95% CI)	P_Q_	OR(95% CI)	P_Q_	OR(95% CI)	P_Q_	OR(95% CI)	P_Q_
Overall	4564/3985	1.54[1.00,2.39]	0.45	1.10[0.96,1.26]	0.26	1.86[1.42,2.43]	0.11	2.34[2.07,2.65]	0.18	2.89[1.87,4.47]	0.98	1.07[0.90,1.26]^a^	0.0005	2.11[1.91,2.35]	0.27
SA	3463/3079	1.67[0.86,3.23]	0.86	1.12[0.97,1.31]	0.98	1.98[1.38,2.85]	0.85	2.23[1.94,2.57]	0.12	2.41[1.43,4.08]	0.95	1.13[0.99,1.29]	0.92	2.07[1.83,2.33]	0.42

SS: sample size.

### Quantitative Synthesis

**Figure 2:**
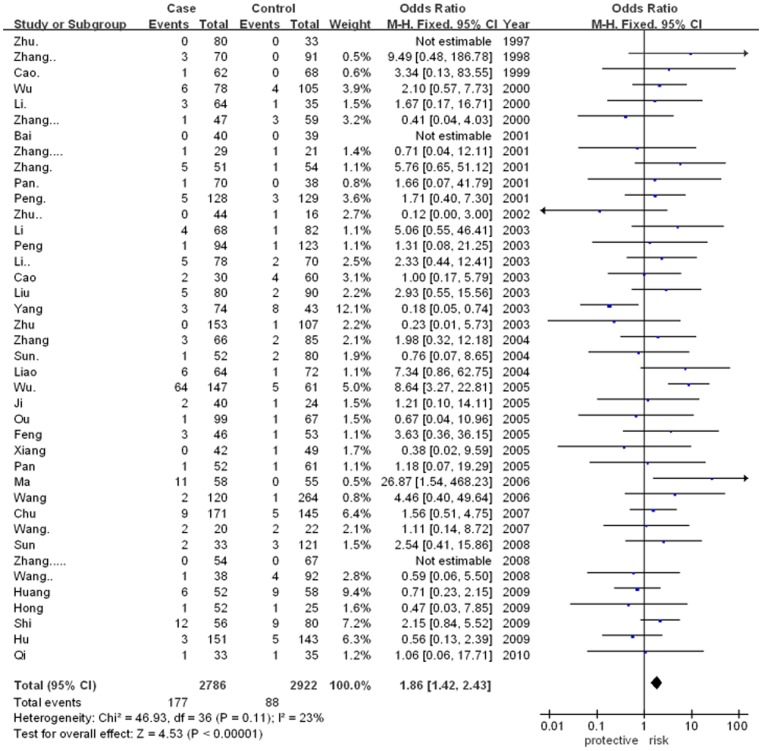
Forest plot for ApoE gene polymorphism and CAD risk in the genetic model ofε2/ε4 vs. ε3/ε3.

**Figure 3:**
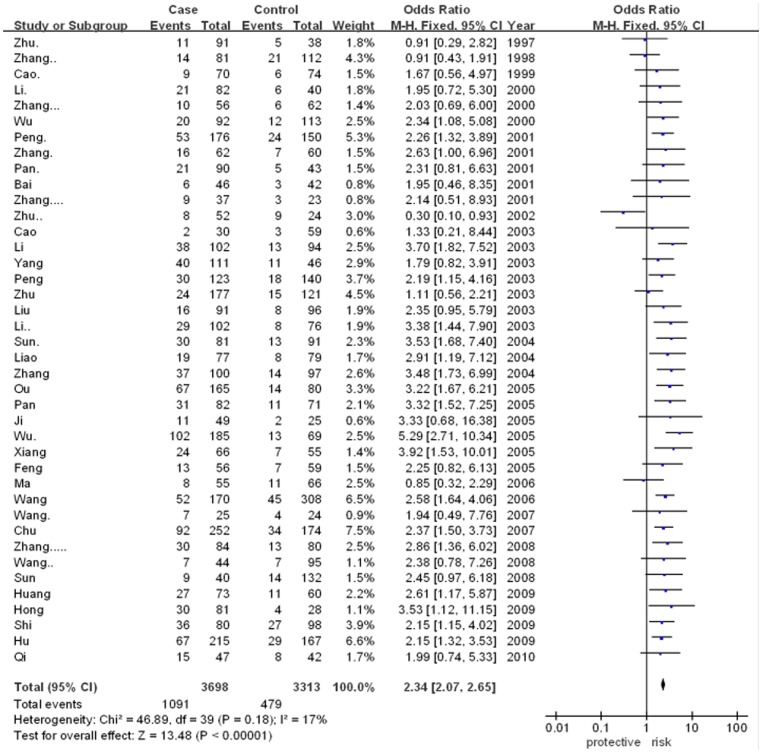
Forest plot for ApoE gene polymorphism and CAD risk in the genetic model ofε3/ε4 vs. ε3/ε3.

**Figure 4:**
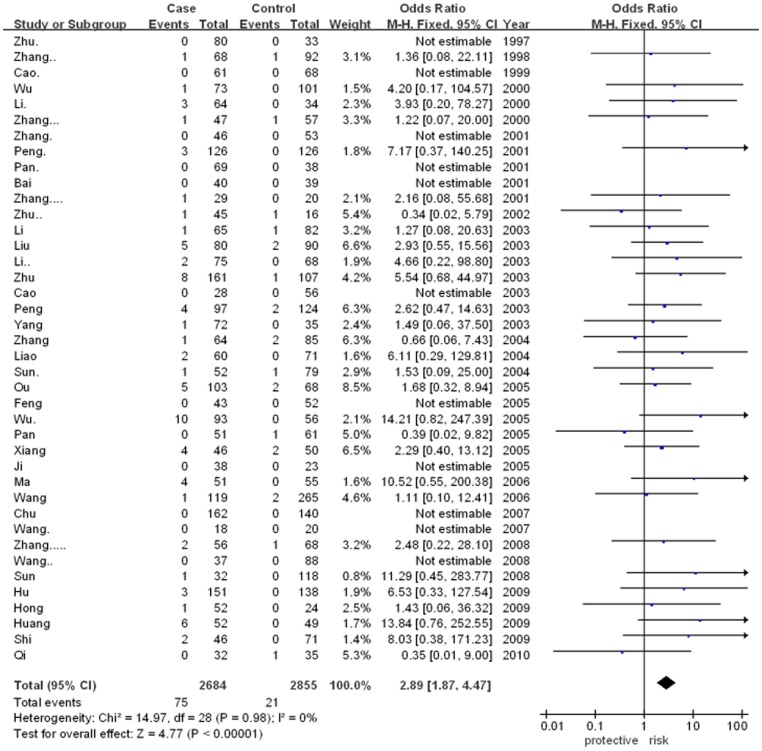
Forest plot for ApoE gene polymorphism and CAD risk in the genetic model ofε4/ε4 vs. ε3/ε3.

**Figure 5:**
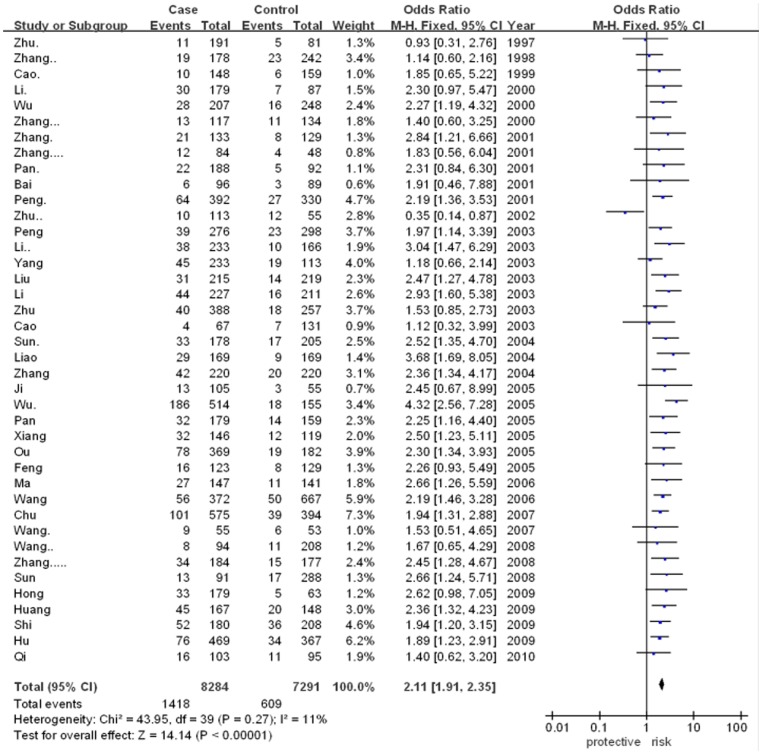
Forest plot for ApoE gene polymorphism and CAD risk in the genetic model ofε4 allele vs. ε3 allele.

The meta-analysis showed that there was significant association between ApoE gene polymorphism and CAD risk (for ε2/ε4 vs. ε3/ε3: OR = 1.86, 95% CI = 1.42–2.43, p<0.00001; for ε3/ε4 vs. ε3/ε3: OR = 2.34, 95% CI = 2.07–2.65, p<0.00001; for ε4/ε4 vs. ε3/ε3: OR = 2.89, 95% CI = 1.87–4.47, p<0.00001). However, there was no significant association in other genetic models. The results were as followed: ε2/ε2 vs. ε3/ε3 (for OR = 1.54, 95% CI = 1.00–2.39, p = 0.05) and ε2/ε3 vs. ε3/ε3 (for OR = 1.10, 95% CI = 0.96–1.26, p = 0.17). In addition, the allele-based contrasts also revealed a statistically significant OR for the contrast of genetic model of ε4 allele vs. ε3 allele (for OR = 2.11, 95% CI = 1.91–2.35, p<0.00001) but not for the genetic model of ε2 allele vs. ε3 allele (for OR = 1.07, 95% CI = 0.90–1.26, p = 0.46).The main results of meta-analysis were shown in Fig. 2, 3, 4, 5 and Table 2, respectively.

### Sensitivity Analysis

The sensitivity analysis was performed with the studies conforming to HWE. Ten studies [17], [26], [29], [31], [37], [39], [46], [47], [49], [51] without HWE were excluded (P<0.05). The corresponding pooled ORs were not materially altered in overall comparisons. The results of the sensitivity analysis were shown in Table 2.

### Heterogeneity Analysis

Significant between-study heterogeneity existed in the genetic model of ε2 allele vs. ε3 allele (for P _Q_ = 0.0005, I^2^ = 48%). In contrast, the other six genetic models did not present significant heterogeneity (for ε2/ε2 vs. ε3/ε3: P_ Q_ = 0.45, I^2^ = 1%; for ε2/ε3 vs. ε3/ε3: P _Q_ = 0.26, I^2^ = 12%; for ε2/ε4 vs. ε3/ε3: P _Q_ = 0.11, I^2^ = 23%; for ε3/ε4 vs. ε3/ε3: P _Q_ = 0.18, I^2^ = 17%; for ε4/ε4 versus ε3/ε3: P _Q_ = 0.98, I^2^ = 0%; for ε4 allele versus ε3 allele: P _Q_ = 0.27, I^2^ = 11%). To detect the source of heterogeneity, we firstly performed the sensitivity analysis by limiting the meta-analysis to studies conforming to HWE, and the heterogeneity was effectively removed from the genetic model of ε2 allele vs. ε3 allele (for ε2 allele vs. ε3 allele: P _Q_ = 0.92, I^2^ = 0%) (Table 2).We next created a Galbraith plot to graphically assess the source of heterogeneity. Four studies were identified as the main contributor of heterogeneity [26], [37], [43], [49] (Fig. S1). After excluding the outlier studies, the heterogeneity was also effectively removed (for ε2 allele vs. ε3 allele: P _Q_ = 1.00, I^2^ = 0%) (Fig. S2).

### Publication Bias

**Figure 6:**
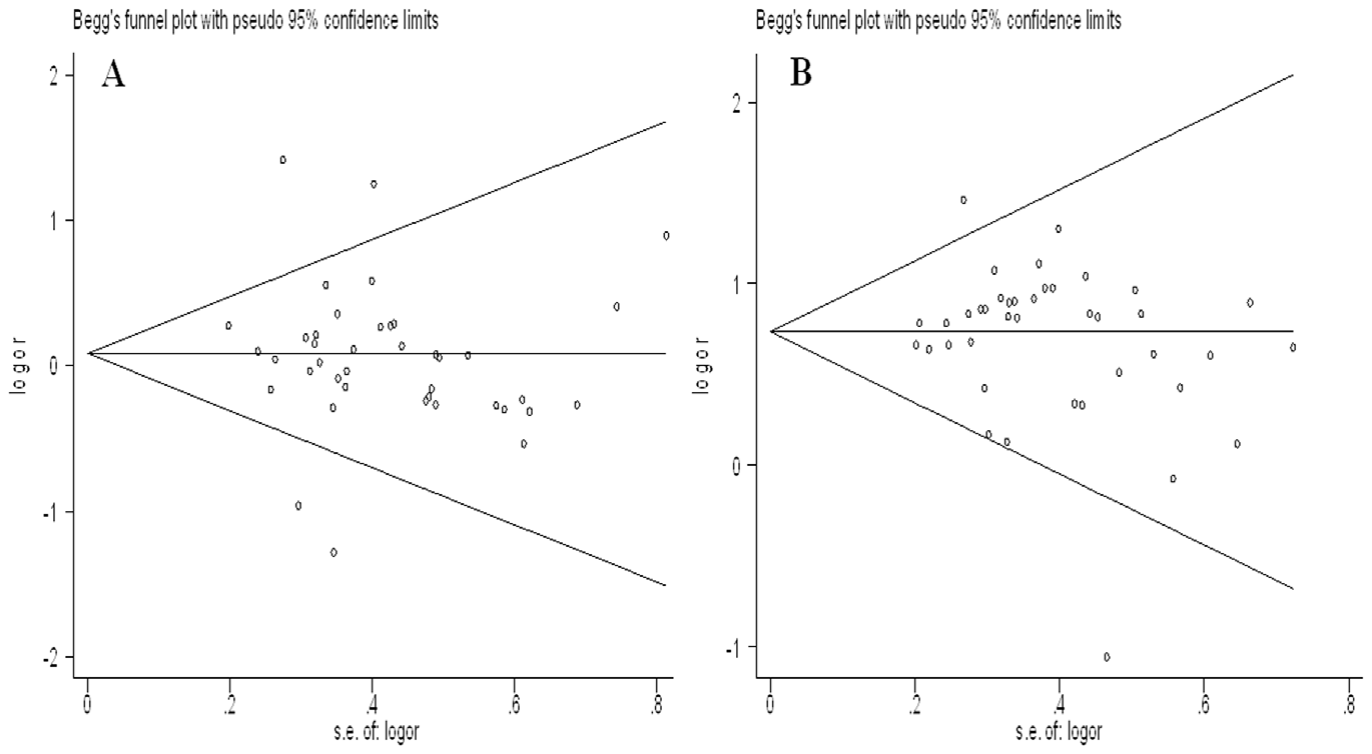
Funnel plots for ApoE gene polymorphism and CAD risk. A: ε2 allele vs. ε3 allele B: ε4 allele vs. ε3 allele.

Begg’s funnel plot and Egger’s regression test were performed to assess the publication bias. As shown in Fig. 6 (eg. Fig. 6A for ε2 allele vs. ε3 allele and Fig. 6B for ε4 allele vs. ε3 allele), no obvious asymmetry was observed in any genetic model. Furthermore, the results of Egger’s regression test still did not provide any evidence for publication bias (p = 0.053 for ε2/ε2 vs. ε3/ε3, p = 0.060 for ε2/ε3 vs. ε3/ε3, p = 0.344 for ε2/ε4 vs. ε3/ε3, p = 0.205 for ε3/ε4 vs. ε3/ε3, p = 0.063 for ε4/ε4 vs. ε3/ε3, p = 0.457 for ε2 allele vs. ε3 allele,and p = 0.288 for ε4 allele vs. ε3 allele).

## Discussion

CAD is a multi-factorial and polygenic disorder disease which is thought to be the result of interactions between complex gene-gene and gene-environment. The association between ApoE gene polymorphism and the risk of CAD has been widely studied, but the results remain inconclusive [14]–[53]. In addition, the credibility of results from a single case-control study is questionable due to too small sample size of the study populations. As suggested, to generate robust data, a much larger sample size in each group might be required [54]. By increasing the sample size, the meta-analysis has the potential to detect small effects in human genetic association studies. To clarify the association between ApoE gene polymorphism and CAD risk, we performed this meta-analysis to examine the allele, genotype of ApoE gene polymorphism in cases and controls.

The present meta-analysis of 40 studies, focusing on only Chinese populations and including 4,564 CAD cases and 3,985 controls, provided most comprehensive analysis on the relationship between ApoE gene polymorphism and CAD risk. Our meta-analysis showed that the risk of developing CAD in ε4 allele carriers was 2.11-fold higher than individuals without ε4 allele. Moreover, the individuals with ε2/ε4 genotype, ε3/ε4 genotype and ε4/ε4 genotype had a significantly higher risk for developing CAD (for OR = 1.86, OR = 2.34 and OR = 2.89) compared to those with ε3/ε3 genotype. Therefore, it is reasonable to assume that the ε4 allele of ApoE is an independent risk factor for the development of CAD in Chinese population. In addition, considering the results produced from genetic association case-control studies may be spurious when the genotype distribution of controls deviates from HWE [55], we also performed sensitivity analysis restricted to the studies conforming to HWE. We found that the corresponding pooled ORs were not materially altered in all genetic models, indicating that the study without HWE should not be considered as a factor influencing the overall results. A previous meta-analysis of worldwide studies including 48 studies has provided evidence that ε4 allele of ApoE is a risk factor for the development of CAD [7]. Consistently, the present meta-analysis obtained the same conclusion.

Heterogeneity is a potential problem that may affect the interpretation of the results. Significant heterogeneity existed in meta-analyses of ε2 allele vs. ε3 allele. Common reasons of heterogeneity may attribute to the diversity in design, sample-sizes, methods of genotyping and inclusion criteria. We found that the studies without HWE were the main factor contributing to initial heterogeneity. When we performed sensitivity analysis by limiting the meta-analysis to studies conforming to HWE, the heterogeneity was effectively removed. In addition, the studies of Yang et al., Wu et al., Ma et al. and Huang et al. were identified as the main contributor of heterogeneity by using Galbraith plot [26], [37], [43], [49]. After excluding the outlier studies, the heterogeneity was also effectively removed and the pooled OR was not materially altered in the genetic model of ε2 allele vs. ε3 allele.

Publication bias did not exist in the overall comparisons, indicating that the results of present meta-analysis were statistically robust. However, only full text articles published in English and Chinese were included in this meta-analysis, missing some eligible studies which were unpublished or reported in other languages. In this case, some inevitable publication bias might exist in the results, although neither the funnel plots nor Egger’s regression test indicated obvious publication bias in the present meta-analysis. It might influence the interpretation of our final results supporting the role of ApoE ε4 allele in CAD. Therefore, projections from the literature of who is at risk for ApoE gene attributable CAD and who would benefit from ApoE gene-targeted therapies should be approached with caution.

For better interpreting the results, some limitations of this meta-analysis should be acknowledged. First, between-study heterogeneity in our analysis should be noted, which may affect the results of the present meta-analysis. Second, subgroup analysis was not performed by the factors such as gender, age and smoking habits because insufficient data could be extracted from the primary article. Furthermore, we also did not perform subgroup analysis by the subtype of CAD (early-onset CAD and late-onset CAD) due to only three studies clearly described the subtype of CAD [28], [32], [45], and the sample sizes of these three studies are really small (212 early-onset cases and 536 controls) and underpowered and thus, unable to provide a definite answer even in the case where a true association exists. Third, some limitations of meta-analysis are inherent (including this one), such as their retrospective nature that is subject to the methodological deficiencies of the included studies. Moreover, China is a multi-ethnic country. We were unable to perform subgroup analysis by ethnic group beacuse the studies in Chinese minority are relatively few and constitute small sample sizes.

In conclusion, our meta-analysis of 40 studies suggests that ApoE ε4 allele is associated with increased CAD risk in Chinese population. Further studies with large sample size, especially in subgroup analysis of Chinese minority, were needed to confirm our findings.

## Supporting Information

Figure S1 **Galbraith plot for ApoE gene polymorphism and CAD risk (ε2 allele vs. ε3 allele).**
(TIF)

Figure S2 **Forest plot for ApoE gene polymorphism and CAD risk after excluding the outlier studies (ε2 allele vs. ε3 allele).**
(TIF)

Table S1 **PRISMA 2009 checklist.**
(DOC)
