# Supplementary figures and images for: Association between Apolipoprotein E Gene Polymorphism and the Risk of Coronary Artery Disease in Chinese Population: Evidence from a Meta-Analysis of 40 Studies
Source: PLoS One. 2013 Jun 24;8(6):e66924. doi: 10.1371/journal.pone.0066924 (PMC3691255; doi:10.1371/journal.pone.0066924)

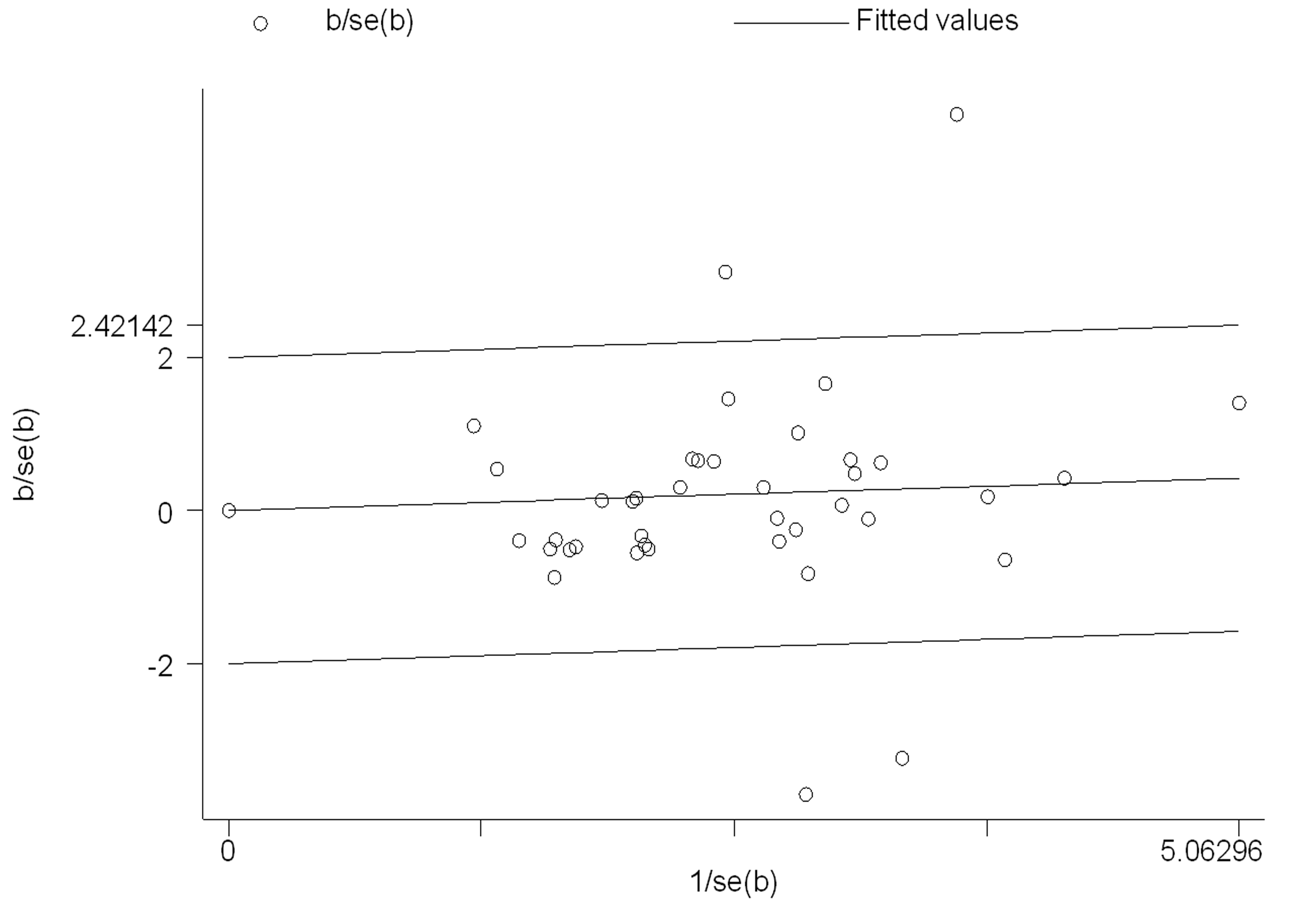

Supplement: Figure S1 — Galbraith plot for ApoE gene polymorphism and CAD risk (ε2 allele vs. ε3 allele). (TIF) [file pone.0066924.s001.tif]

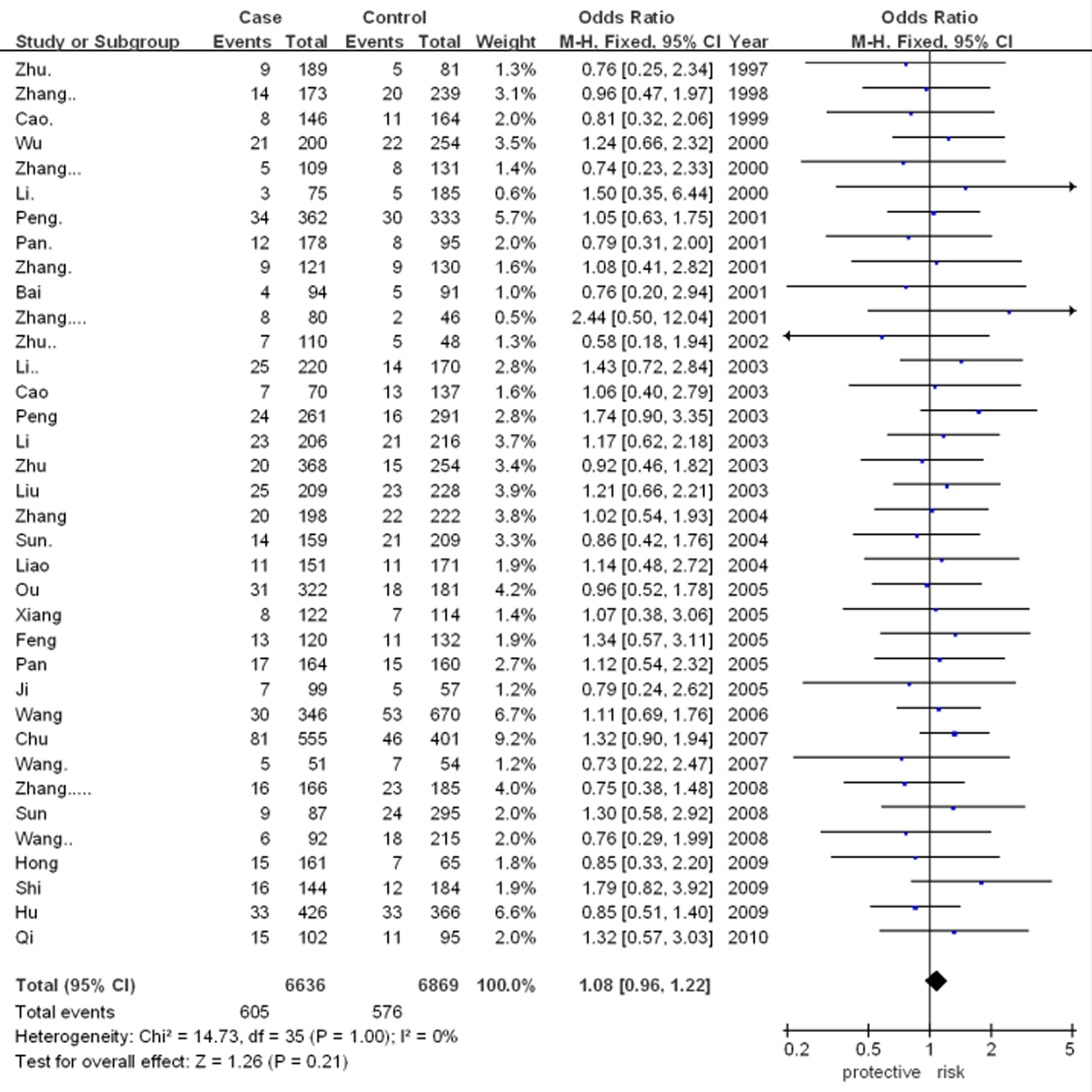

Supplement: Figure S2 — Forest plot for ApoE gene polymorphism and CAD risk after excluding the outlier studies (ε2 allele vs. ε3 allele). (TIF) [file pone.0066924.s002.tif]
