# Supplementary material for: Association between Apolipoprotein E Gene Polymorphism and the Risk of Coronary Artery Disease in Chinese Population: Evidence from a Meta-Analysis of 40 Studies
Source: PLoS One. 2013 Jun 24;8(6):e66924. doi: 10.1371/journal.pone.0066924 (PMC3691255; doi:10.1371/journal.pone.0066924)
Supplement: Table S1 — PRISMA 2009 checklist. (DOC) [file pone.0066924.s003.doc]

| **Section/topic** | **#** | **Checklist item** | **Reported on page #** |
| --- | --- | --- | --- |
| **TITLE** | | |  |
| Title | 1 | Association between apolipoprotein E gene polymorphism and the risk of coronary artery disease in Chinese population: Evidence from a meta-analysis of 40 studies | 1 |
| **ABSTRACT** | | |  |
| Structured summary | 2 | **Background:** Epidemiological studies have evaluated the association between apolipoprotein E (ApoE) gene polymorphism and coronary artery disease (CAD) risk which developed inconsistent conclusions. To derive a more precise estimation of the relationship in Chinese population, we performed this meta-analysis.  **Methods:** Databases, including PubMed, EMbase, Web of Science, CBMdisc and CNKI, were searched to get the genetic association studies. Additionally, hand searching of the references of identiﬁed articles were performed. All the statistical tests were performed using Review Manager 5.1.2 and Stata 11.0.  **Results:** We identiﬁed a total of 40 studies, including 4,564 CAD cases and 3,985 controls. The results showed evidence for significant association between ApoE ε4 allele and CAD risk (for ε2/ε4 vs. ε3/ε3: OR = 1.86, 95% CI = 1.42-2.43, p﹤0.00001; for ε3/ε4 vs. ε3/ε3: OR = 2.34, 95% CI = 2.07-2.65, p﹤0.00001; for ε4/ε4 vs. ε3/ε3: OR = 2.89, 95% CI = 1.87-4.47, p﹤0.00001; for ε4 allele vs. ε3 allele: OR = 2.11, 95% CI = 1.91-2.35, p﹤0.00001).  **Conclusions:** The present meta-analysis suggests an association between ApoE ε4 allele and increased risk of CAD in Chinese population. However, due to the small sample size in most of the included studies and the selection bias existed in some studies, the results should be interpreted with caution. | 1-2 |
| **INTRODUCTION** | | |  |
| Rationale | 3 | ApoE is a plasma protein involved in the lipid metabolism and participates in the transports of cholesterol and triglyceride [1-3]. ApoE gene polymorphism have been found to affect ApoE gene transcription and the serum levels of cholesterol and triglyceride [5,6], thus changing the progression of atherosclerosis, which is the main underlying pathology of CAD. Recently, numerous molecular epidemiological studies have focused on the association between apolipoprotein E (ApoE) gene polymorphism and CAD risk, and indicated that ApoE ε4 allele exerts an important role in the development of CAD. | 2-3 |
| Objectives | 4 | Recently, a variety of molecular epidemiological studies have focused on the relationship between ApoE gene polymorphism and CAD risk. However, results in different studies have been inconsistent. A meta-analysis of worldwide studies including 48 studies has provided evidence that the ε4 allele of ApoE was a risk factor for the development of CAD [7]. Considering that potential ethnic difference might be associated with the distribution of genotypes, we conducted a meta-analysis by collecting and sorting the previously published studies in Chinese population. | 2-3 |
| **METHODS** | | |  |
| Protocol and registration | 5 | No protocol and registration. |  |
| Eligibility criteria | 6 | Studies included in the current meta-analysis had to meet the following criteria: (1) studies on the relationship between ApoE gene polymorphism and CAD; (2) case-control studies using either a hospital-based or a population-based design; (3) studies with full text articles; (4) sufficient data for estimating an odds ratio (OR) with 95% confidence interval (CI); (5) not republished data. | 4 |
| Information sources | 7 | We performed a comprehensive computer-based searche of PubMed, Embase, Web of Science, Chinese Biomedical Literature analysis and retrieval system for compact disc (CBMdisc) and China National Knowledge Infrastructure (CNKI) (up to November 28, 2012) to identify studies analyzing the association of ApoE gene polymorphism with CAD. Additionally, the references of the eligible articles were searched to identify citations to other studies that were not identiﬁed initially. | 3-4 |
| Search | 8 | The following keywords were used for searching: “apolipoprotein E” OR “ApoE” AND “polymorphism” OR “mutation” OR “variant” OR “variation” OR “genotype” AND “coronary artery disease” OR “CAD” OR “coronary heart disease” OR “CHD” OR “ischemic cardiovascular disease” OR “atherosclerosis”. The equivalent Chinese terms were used in the Chinese databases. Additionally, hand searches for related articles were also performed. | 4 |
| Study selection | 9 | The study selection process is detailed in Figure. 1. A total of 40 studies were included in the final meta-analysis according to the inclusion criteria, containing 4,564 CAD cases and 3,985 controls. | 6 |
| Data collection process | 10 | Data was independently extracted by two authors of this article (Yin YW and Hu AM), and the result was reviewed by a third author (Sun QQ). | 4-5 |
| Data items | 11 | For each study, information was extracted including first author, year of publication, area of study population, source of controls, total numbers of cases and controls, genotyping methods, and distribution of genotypes and alleles in cases and controls, respectively. In addition, evidence of Hardy-Weinberg equilibrium was also collected (HWE, p < 0.05 of HWE was considered significant). | 5 |
| Risk of bias in individual studies | 12 | Two authors (Liu HL and Wang Q) of this article independently assessed the quality of included studies using the Newcastle-Ottawa Scale (NOS). The third author (Hou ZZ) examined the results, and disagreement was resolved by discussion. | 5 |
| Summary measures | 13 | The principal summary measures are odds ratios(ORs) and 95% confidence intervals (CIs). | 5 |
| Synthesis of results | 14 | The pooled ORs were estimated for seven genetic models (ε2/ε2 vs. ε3/ε3, ε2/ε3 vs. ε3/ε3, ε2/ε4 vs. ε3/ε3, ε3/ε4 vs. ε3/ε3, ε4/ε4 vs. ε3/ε3, ε2 allele vs. ε3 allele and ε4 allele vs. ε3 allele). Heterogeneity between studies was formally tested by using Cochran’s Q statistic and considered statistically signiﬁcant when p＜0.10. Heterogeneity was also measured with I2 statistic (I^2^>50% indicated evidence of heterogeneity). The fixed-effects model was used in the absence of between-study heterogeneity; otherwise the random-effects model was used. | 5-6 |

Page 1 of 2

| **Section/topic** | **#** | **Checklist item** | **Reported on page #** |
| --- | --- | --- | --- |
| Risk of bias across studies | 15 | An estimate of potential publication bias was carried out by Begg’s funnel plot and Egger’s regression test (p < 0.05 was considered representative of statistically signiﬁcant publication bias) | 6 |
| Additional analyses | 16 | Galbraith plot was used to detect the potential sources of heterogeneity. Sensitivity analysis was conducted by limiting the meta-analysis to studies conforming to HWE. | 6 |
| **RESULTS** | | |  |
| Study selection | 17 | The study selection process is detailed in Figure. 1. A total of 40 studies were included in the final meta-analysis according to the inclusion criteria, containing 4,564 CAD cases and 3,985 controls. | 6 |
| Study characteristics | 18 | Table 1 shows the studies included in the meta-analysis and their main characteristics. | 6 |
| Risk of bias within studies | 19 | The NOS results were shown in Table 1. The results showed that the average score was 7.85, which indicated that the methodological quality was generally good. | 6 |
| Results of individual studies | 20 | The main results of individual studies were shown in Fig 2, Fig 3, Fig 4, and Fig 5. (Fig. 2 , Fig. 3, Fig. 4, and Fig.5: Forest plots for ApoE gene polymorphism and CAD risk in different genetic models.). | 7 |
| Synthesis of results | 21 | The meta-analysis showed that there was significant association between ApoE gene polymorphism and CAD risk (for ε2/ε4 vs. ε3/ε3: OR = 1.86, 95% CI = 1.42-2.43, p﹤0.00001; for ε3/ε4 vs. ε3/ε3: OR = 2.34, 95% CI = 2.07-2.65, p﹤0.00001; for ε4/ε4 vs. ε3/ε3: OR = 2.89, 95% CI = 1.87-4.47, p﹤0.00001). The main results of meta-analysis were shown in Table 2, Fig. 2 , Fig. 3, Fig. 4, and Fig. 5. | 7 |
| Risk of bias across studies | 22 | Begg’s funnel plot and Egger’s regression test were performed to assess the publication bias. As shown in Fig. 6 (eg. Fig. 6A for ε2 allele vs. ε3 allele and Fig. 6B for ε4 allele vs. ε3 allele), no obvious asymmetry was observed in any genetic model. Furthermore, the results of Egger’s regression test still did not provide any evidence for publication bias (p = 0.053 for ε2/ε2 vs. ε3/ε3, p = 0.060 for ε2/ε3 vs. ε3/ε3, p = 0.344 for ε2/ε4 vs. ε3/ε3, p = 0.205 for ε3/ε4 vs. ε3/ε3, p = 0.063 for ε4/ε4 vs. ε3/ε3, p = 0.457 for ε2 allele vs. ε3 allele,and p = 0.288 for ε4 allele vs. ε3 allele) | 8 |
| Additional analysis | 23 | **Sensitivity analysis:** The sensitivity analysis was performed with the studies conforming to HWE. Ten studies without HWE were excluded (P<0.05). The corresponding pooled ORs were not materially altered in overall comparisons. The results of the sensitivity analysis were shown in Table 2.  **Heterogeneity analysis:** Signiﬁcant between-study heterogeneity existed in the genetic model of ε2 allele vs. ε3 allele (for P _Q_ = 0.0005, I^2^= 48%). In contrast, the other six genetic models did not present significant heterogeneity (for ε2/ε2 vs. ε3/ε3: P _Q_ = 0.45, I^2^ = 1% ; for ε2/ε3 vs. ε3/ε3: P _Q_ = 0.26, I^2^= 12% ; for ε2/ε4 vs. ε3/ε3: P _Q_ = 0.11, I^2^= 23% ; for ε3/ε4 vs. ε3/ε3: P Q = 0.18, I2 = 17% ;for ε4/ε4 versus ε3/ε3: P Q = 0.98, I2= 0% ; for ε4 allele versus ε3 allele: P _Q_ = 0.27, I^2^ = 11%). To detect the source of heterogeneity, we firstly performed the sensitivity analysis by limiting the meta-analysis to studies conforming to HWE, and the heterogeneity was effectively removed from the genetic model of ε2 allele vs. ε3 allele (for ε2 allele vs. ε3 allele: P _Q_ = 0.92, I^2^ = 0%) (Table 2).We next created a Galbraith plot to graphically assess the source of heterogeneity. Four studies were identified as the main contributor of heterogeneity (Fig. S1). After excluding the outlier studies, the heterogeneity was also effectively removed (for ε2 allele vs. ε3 allele: P _Q_ = 1.00, I^2^ = 0%) ( Fig. S2). | 7-8 |
| **DISCUSSION** | | |  |
| Summary of evidence | 24 | In the present meta-analysis, we examined ApoE gene polymorphism and its relationship with the risk of CAD in seven genetic models. The results ((for ε2/ε4 vs. ε3/ε3: OR = 1.86, 95% CI = 1.42-2.43, p﹤0.00001; for ε3/ε4 vs. ε3/ε3: OR = 2.34, 95% CI = 2.07-2.65, p﹤0.00001; for ε4/ε4 vs. ε3/ε3: OR = 2.89, 95% CI = 1.87-4.47, p﹤0.00001; for OR = 2.11, 95% CI = 1.91-2.35, p﹤0.00001)) showed that the individuals with ApoE gene ε4 allele had a significantly higher risk for developing CAD compared to those with ε2 allele and/or ε3 allele. The above results suggested that ApoE gene ε4 allele may be a risk factor for CAD. | 9 |
| Limitations | 25 | **Limitations:** First, between-study heterogeneity in our analysis should be noted, which may affect the results of the present meta-analysis. Second, subgroup analysis was not performed by the factors such as gender, age and smoking habits because insufficient data could be extracted from the primary article. Furthermore, we also did not perform subgroup analysis by the subtype of CAD (early-onset CAD and late-onset CAD) due to only three studies clearly described the subtype of CAD [28,32,45], and the sample sizes of these three studies are really small (212 early-onset cases and 536 controls) and underpowered and thus, unable to provide a definite answer even in the case where a true association exists. Third, some limitations of meta-analysis are inherent (including this one), such as their retrospective nature that is subject to the methodological deficiencies of the included studies. Moreover, China is a multi-ethnic country. We were unable to perform subgroup analysis by ethnic group beacuse the studies in Chinese minority are relatively few and constitute small sample sizes. | 11 |
| Conclusions | 26 | **Conclusion：**In conclusions, our meta-analysis of 40 studies suggests that ApoE ε4 allele is associated with increased CAD risk in Chinese population. Further studies with large sample size, especially in subgroup analysis of Chinese minority, were needed to confirm our findings. | 12 |
| **FUNDING** | | |  |
| Funding | 27 | No current external funding sources for this study. |  |

*From:*  Moher D, Liberati A, Tetzlaff J, Altman DG, The PRISMA Group (2009). Preferred Reporting Items for Systematic Reviews and Meta-Analyses: The PRISMA Statement. PLoS Med 6(6): e1000097. doi:10.1371/journal.pmed1000097

For more information, visit: **www.prisma-statement.org**.

Page 2 of 2
